# Post‐mortem investigation into a death involving doping agents: The case of a body builder

**DOI:** 10.1002/dta.3350

**Published:** 2022-08-14

**Authors:** Donata Favretto, Giulia Stocchero, Roberto Pertile, Raffaella Stimamiglio, Antonello Cirnelli, Maddalena Galeazzi

**Affiliations:** ^1^ Legal Medicine and Toxicology University Hospital of Padua Padua Italy; ^2^ Toxicology Sector Provincial Health Services Agency Trento Italy; ^3^ Specialist in Legal Medicine in Portogruaro Portogruaro Italy; ^4^ School of Specialisation in Legal Medicine University Hospital of Padua Padua Italy

## Abstract

**Introduction:**

A young male was found dead on the bed of a hotel room. He was expected to take part in a bodybuilding competition the day after. During the site inspection, drugs of different types were found. The next day, an autopsy was performed. The evidence of cardiomegaly with organ congestion involving lung, liver, kidneys, adrenal glands, spleen and brain was confirmed by both the autoptic and the histopathological exam. However, the cause of death needed to be investigated.

**Methods:**

A thorough toxicological investigation was undertaken by gas chromatography–mass spectrometry (GC‐MS), liquid chromatography‐high resolution mass spectrometry (LC‐HRMS) and liquid chromatography–tandem mass spectrometry (UPLC‐MS/MS) on samples of urine, blood and hair.

**Results and discussion:**

Clenbuterol, a long‐acting selective beta_2_ agonist, was found in both blood (1 ng/ml) and urine (1 ng/ml), and evidence of its use was provided by the analysis of the 3‐cm hair (25 pg/mg). The main metabolite of drostanolone (2 alpha‐methyl‐androsterone), an anabolic steroid, was found in the urine (202 ng/ml), where an increased ratio of testosterone/epitestosterone (T/E = 11) emerged. Due to the results of the hair analysis, a long‐term use of various anabolic steroids was supposed. The integrated analysis of the results and the absence of other possible causes (such as trauma or cardiac conduction anomalies) led to the identification of the abuse of doping substances as the underlying cause of death.

**Conclusion:**

Hair analysis has proven to be crucial in identifying drug misuse and the contributing cause of death.

## 1 INTRODUCTION

The wide class of anabolic agents consists of (1) exogenous anabolic androgenic steroids (AAS), including, for instance, stanozolol, metandienone and oxandrolone; endogenous AAS such as testosterone, dehydroepiandrosterone and androstenedione and (2) other anabolic agents including clenbuterol, selective androgen receptor modulators, tibolone, zeranol and zilpaterol.

Clenbuterol can be classified as a long‐acting selective beta_2_ agonist^1^ that maintains its main therapeutic indication in the treatment of bronchial asthma.^2^ Its established effects on the relaxation of bronchial smooth muscle, the decrease of mucus production and the increase of mucociliary transport in the airways support its clinical use. Further studies have shown its antiallergic activity against anaphylactic‐induced asthmatic attacks in its capacity to stabilise the membrane of mast cells and basophils. In Italy, clenbuterol is available in the form of syrup or in tablets dosed at 20 μg, with the maximum recommended daily dose fixed at 40 μg.^3^ In other countries, injectable solutions and aerosols are available.

The wide category of AAS includes more than 100 active principles, most of which lack governmental regulatory health authorities' approval for human therapeutic use (e.g., discontinued drugs, designer drugs and substances approved only for veterinary use), and only a few of which have therapeutic indications in animals (stanozolol) or in humans (e.g., testosterone), including hypogonadism, hormone sensitive neoplasms, medullary aplasia and osteoporosis.^4^ Due to their impact on protein synthesis,^5^ muscle growth and erythropoiesis, these substances have shown benefits in a variety of conditions, including HIV‐related muscle wasting, muscle dystrophies, severe burn injuries, bone marrow failure, hereditary angioedema and growth retardation in children.^4^ The anabolic‐androgenic effects, which can be identified in the increase of mean muscle mass^6^ and enhanced erythropoiesis, the stimulation of aggression and the decrease of the training‐induced fatigue, sustain their widespread use among bodybuilders and athletes. The amplification of therapeutic effects, due to long‐term use, leads to toxicity in term of high blood pressure with hypertrophy and coronary disease, liver damage, increased risk of prostate cancer, decreased libido, dermatological changes and metabolic imbalances.

A variety of side effects can occur when anabolic steroids are misused, ranging from mild effects to ones that are harmful or even life‐threatening. Most are reversible if the user stops taking the drugs. However, others may be permanent or semipermanent.^7^ The main side effects on the human body regard the cardiovascular system (increase of blood pressure, blood clots, heart attack and stroke),^8^ the hormonal system (decreased sperm production, testicular cancer and excessive body hair growth), the liver (peliosis hepatis, and tumours), the musculoskeletal system (tendon injury and short stature), the skin (acne and cysts) and the immune system (hepatitis and HIV). Most data on the long‐term effects of anabolic steroids in humans come from case reports rather than from formal epidemiological studies. Serious and life‐threatening adverse effects may be underreported, especially since they may occur many years later. One review found 19 deaths in published case reports related to anabolic steroid use between 1990 and 2012; however, many steroid users also used other drugs, making it difficult to show that the anabolic steroid use caused these deaths. One animal study found that exposing male mice to steroid doses comparable to those taken by human athletes for one fifth of their lifespan caused a high frequency of early deaths.^7^

The authors present here a fatal case of a bodybuilder involving the use of clenbuterol and AAS.

## 2 CASE REPORT

The day before a bodybuilding regional competition, a 24‐year‐old male was found inanimate by his girlfriend, with whom he shared the room. She reported that they had spent the night before in a restaurant drinking some alcohol and went to sleep at 1:00 a.m. He went to the toilet only once during the night. At 6.00 a.m., she found the corpse of her boyfriend laying on the bed next to her. According to her, he habitually used thyroid medications and diuretics for sport activities, but he had not assumed any drugs before bedtime. ‘As far as I know’, she said, ‘he didn't suffer from diseases of any type’. Police inspection of the room revealed the presence of many drugs, one package of each, later identified as Diuresix® (torasemide)®, Monores® (clenbuterol hydrochloride), Eutirox® (levothyroxine sodium), Halotestin® (fluoxymesterone) and Lasix® (furosemide). Two empty disposable syringes were collected as well. The autopsy was carried out by the judicial authority the day after at the Pathological Anatomy Unit of Padua University, where samples were collected for toxicological ascertainments. Generalised organ congestion, including of the meningeal veins, spleen and the liver itself, was revealed by the macroscopic and the microscopic analysis. The lungs were affected by a massive edema with haemorrhagic extravasation, while the heart was characterised by a cardiomegaly with nonobstructive concentric intimal hyperplasia. Femoral and cardiac blood, urine, bile, gastric contents, vitreous humour and hair as well as fragments of kidney, brain and liver were collected for toxicology investigations.

## 3 MATERIAL AND METHODS

### 3.1 Toxicological initial screening

The case specimens were subjected to a laboratory post‐mortem toxicological screening battery. Ethanol and volatiles were tested in blood and urine by a head space gas chromatography‐flame ionisation detector on an Agilent 6890 system using an in‐house validated procedure. An immunoassay test for pharmaceuticals and abused drugs was carried out on blood and urine using Siemens kits and following the recommendations of the manufacturer. A general unknown screening was performed on blood and urine by means of LC‐HRMS on an Orbitrap LTQ (Thermofisher), and a target screening was performed by UPLC‐MS/MS on a Xevo TQS (Waters). Subsequently, the quantification of clenbuterol in the blood was realised by a multiple reaction monitoring method using UPLC/MS‐MS; clenbuterol and AAS steroids in the hair were identified and quantified by LC‐HRMS, while clenbuterol and AAS in the urine were determined by GC‐MS. Diuretics were not detected in the urine or blood.

### 3.2 Urine/blood sample‐preparation for steroids

The urine/blood sample preparation for steroids consists of different phases: solid phase extraction (SPE) on C18 column, enzymatic hydrolysis, liquid‐liquid extraction, final derivatisation and analysis with GC‐MS. The SPE column, previously activated with water and methanol, was added with 5 ml of urine mixed with methyltestosterone and 19‐noretiocolanolone‐d4. After water washing, the elution was performed with methanol; the eluate was dried under nitrogen current, added with phosphate buffer (pH = 7) and hydrolysed with beta‐Glucuronidase deriving from *Escherichiacoli* at 50°C for 2 h. The mixture was cooled and was added with carbonate/bicarbonate buffer (1:10) in order to obtain a solution adjusted at pH of 8.5–9. Finally, extraction was performed with 5 ml anhydrous pentane. The organic phase was dried and derivatised with a mixture of trimethylsilyltrifluoroacetamide/dithioerythritol/ammonium iodide (MSTFA/DTE/NH_4_I) (1 ml:6 mg:4 mg) at 75°C for 20 min.

### 3.3 Urine/blood analysis

The GC‐MS analysis was carried out using a gas chromatograph Agilent 6890N, a single quadrupole Agilent 5973 MSD detector and an autosampler Agilent 7673. The GC column used for the analysis was an HP‐5MS model (25 μm × 0.25 mm inner diameter × 0.25 μm film thickness). The gas used for transport was helium at flow rate of 0.8 ml/min. The amount of sample injected was 2 μl in splitless mode for 0.8 min. The temperature gradient was the following: 90°C (1 min), 15°C/min, ➔ 200C°, 30°C/min ➔ 300°C (10 min); source temperature: 230°C; injector temperature: 275°C; transfer line temperature: 280°C. The ionisation mode was electron ionisation (70 eV). The acquisition method was multiple ion monitoring, as shown in the supporting information Table S1.

### 3.4 Hair sample‐preparation for steroids

Hair, 3 cm long, was resected from the scalp base and washed twice with 5 ml of Tween 80 (0.1% V/V in distilled water) and 5 ml dichloromethane. After drying, it was ground in a mill, added with internal standards and suspended in 1 ml of phosphate buffer (0.1 M and pH = 5). After an incubation of 30 min at 95°C, the mixture was extracted with 4 ml of n‐heptane. Once centrifuged, the organic supernatant was dried and resumed with 100 μl of water/acetonitrile 80/20. A total of 25 μl was injected into the LC‐HRMS.

### 3.5 LC‐HRMS of hair extract

All analysis were performed using an LTQ‐Orbitrap (Thermofisher, Bremen, Germany) HRMS equipped with an electrospray ionisation source, an auto‐sampler and a Surveyor HPLC pump (Thermofisher, Bremen, Germany). The LC conditions were as follows: Atlantis T3 column (150 μm × 2.1 μm); gradient elution with 10 mM aqueous ammonium phosphate buffer containing 0.1% (v/v) formic acid as mobile phase A and acetonitrile containing 0.1% (v/v) formic acid as mobile phase B. The flow rate was 300 ml/min, and the gradient was programmed as follows: 0–0.5 min at 90% A, 0.5–20 min to 0% A and 20–22 min holds in 0% A. The Orbitrap was operated in full‐scan HRMS. The MS conditions were as follows: positive ionisation mode, full scan from *m*/*z* 100 to 800 (resolution 60,000 at *m/z* 400; scan time 0.45 s); sheath gas, nitrogen, at a flow rate of 55 arbitrary units (AU); auxiliary gas, nitrogen, at a flow rate of 18 AU; vaporiser temperature, 250°C; spray voltage, 4.00 kV; ion transfer capillary temperature, 270°C; maximum injection time 500 ms. Detection of the analytes were based on accurate mass measurements coupled with high resolution (Δm/z ≤ 3 ppm), isotope pattern recognition and retention time correspondence versus a homemade library of 800 compounds of relevant toxicological interest and their metabolites.

## 4 RESULTS AND DISCUSSION

Ethanol and other volatile substances tested negative in blood, while in urine, a low concentration of ethanol was revealed (0.17 g/L). The results concerning anabolising agents are reported in Tables 1, 2, 3.

**TABLE 1:** Analytes concentrations (ng/ml) detected in blood and in urine

Substance	Concentration, blood	Concentration, urine
Clenbuterol	1 ng/ml	1 ng/ml
2‐alpha‐methyl‐androsterone	‐	202 ng/ml

**TABLE 2:** Ratio testosterone/epitestosterone (T/E) from urine values

Ratio^a^	Normal value	Detected value
T/E	≤4	11

^a^ The T/E ratios was determined from the ratios of the corrected chromatographic peak areas.

**TABLE 3:** Analytes concentrations (pg/mg) detected in hair

Substance	Pharmacological category	Concentration pg/mg
Clenbuterol	Long‐acting selective beta_2_‐agonist, nonsteroidal anabolic	25
Stanozolol	AAS	42
Testosterone decanoate	AAS	18
Testosterone cypionate	AAS	28
Testosterone undecanoate	AAS	23
Testosterone propionate	AAS	22
Methandienone	AAS	18
Boldenone propionate	AAS	32
Nor androstenedione	AAS	14
Methenolone enanthate	AAS	18
Trenbolone	AAS	25
Drostanolone	AAS	28

The extremely elevated T/E ratio can be considered as atypical, warranting follow‐up investigations as to testosterone misuse in the living and indicating testosterone misuse in the deceased.^9^ The presence of the testosterone esters in the hair suggests that the alteration of the ratio is due to exogenous drugs. The results arising from the blood and urine indicate a recent intake of clenbuterol that, due to its pharmacokinetic properties (t‐max within 2.5 h lasting for over 6 h after the administration), could be dated back a few hours before death. Clenbuterol is the active component of Monores® tablets that were found in the room. The diuretics contained in Lasix® (furosemide) and Diuresix® (torasemide) were not detected in the biological fluids collected from the deceased. In the event of overdose, it leads to clear collateral effects, such as headache, tachycardia, agitation, tremor and palpitations. Other pathological conditions such as vomiting, anxiety, psychosis, myocardial infarction, ventricular arrhythmias, hypokalaemia, lactic acidosis or rhabdomyolysis are described as well.^3^, ^10^, ^11^ In the international scientific literature, many case reports involving users of clenbuterol‐adulterated heroin, subjects eating contaminated livestock and bodybuilders who wish to gain mass or to lose weight^3^, ^12^, ^13^, ^14^ are described. In fact, clenbuterol has the capacity to enhance the contractile force in some muscle fibres, which supports the widespread use of this substance among bodybuilders. However, its global and anabolic effects, when used for athletic performance purposes, have not been clarified yet. Furthermore, it must be considered that the drugs coming from the illicit market and abused by bodybuilders are not produced under good manufacturing practices and can contain active principles different from those stated in the package since they lack quality control. A true ‘therapeutic’ or ‘toxic’ range of concentrations is difficult to obtain for clenbuterol because analytically confirmed reports of clenbuterol use are rare, due to the very low concentrations that may be found in blood or other biological specimens.^2^ In 12 reports concerning clenbuterol, its blood concentrations ranged from undetectable to 76 ng/ml.^12^ In another series,^15^ clenbuterol serum concentrations ranged from 3 to 38 ng/ml (*n* = 7) and were caused by an assumption of heroin adulterated with clenbuterol. In a recent fatal case,^2^ a 61‐year‐old man was found by his wife laying on the floor after a 15‐year history of anabolic steroids abuse. The toxicologic exams made on the corpse emerged an excessive concentration of clenbuterol in the blood (1.1 ng/ml), in the urine (7.2 ng/ml) and in the hair (23 pg/mg). Considering that after a single dose of 40 mg plasma levels of clenbuterol reach peak values ​​of 0.2 ng/ml within 2.5 h and that after repeated administration of doses of 40 μg twice a day in healthy adults, ​​ average stationary values of 0.5–0.6 ng/ml are reached, with a half‐life of about 30 h, the blood value (1.2 ng/ml) indicated a toxic concentration due to continuous and overdosed use of the substance. In that case, the combination of clenbuterol with the anabolic stanzolol, found in the hair only, was considered synergistic and prodromic to severe cardiac toxicity, with a parallelism between long‐term use documented through hair analysis and heart disease. Therefore, the cause of death was identified in the combined use of clenbuterol with an anabolic androgenic steroid that led to myocardial infarction even in the absence of underlying heart disease.^16^ The presence of an underlying cardiac pathological alteration may be present in the form of dilatative cardiomyopathy, acute liver disease and severe coronary artery disease.^17^, ^18^ Furthermore, in an inclusive retrospective study^19^ of 545 long‐time anabolic steroid users, the incidence of atrial fibrillation was three times higher than in the control group, and the risk of thromboembolism was increased fivefold. Therefore, the consumers of AAS have an increased risk of hospital admission than their nonusing peers, with an overall mortality rate 0.7% higher than the controls and a risk of death of 3.0 (95% CI 1.3–7.0). It is interesting to note that in the case presented in this study, clenbuterol values analogous to those reported by Kintz et al.^2^ were ​​found in fluids and hair. As to the metabolite of drostanolone, found in urine only, its finding is linked to recent use, even if it is not possible to establish a specific time window. AAS, indeed, if administered through an intramuscular or intravenous way, create a deposit from which they are released. Afterwards, they are slowly absorbed, transported through the plasma to different organs, metabolised in the liver and lastly excreted by the kidneys. The concentration of drostanolone metabolite revealed in the urine (202 ng/ml) could be considered high enough to suggest an administration within 1 week. The results of the hair analysis show systematic and repeated exposure to anabolic steroids during the 2 to 4 months before death. The intake of multiple steroid active principles is a common practice detectable in the drug's users. Steroids are often used in patterns called ‘cycling’, which involve taking multiple doses of steroids over a specific period, stopping for a period and starting again. People who misuse steroids can also typically ‘stack’ the drugs, taking two or more different anabolic steroids, mixing oral and/or injectable types and even taking compounds that are designed for veterinary use (e.g., stanozolol). They believe that different steroids interact to produce an effect on muscle size greater than the effects of each drug individually; however, this theory has not been proved scientifically. Another common mode of steroid misuse is referred to as ‘pyramiding’, which typically involves taking a drug in a cycle of 6 to 12 weeks, then reducing its use gradually rather than starting and concluding a cycle abruptly. This is sometimes followed by a second cycle in which the person continues to train without using drugs. Steroid users believe that the pyramid system allows the body to adapt to high doses and that the drug‐free cycle leads to the restoration of the hormonal system. It is interesting to report the existence of a technique called ‘plateauing’, which consists of the integrated or exclusive use of other steroids to avoid the onset of tolerance. However, the effects of pyramiding, cycling and plateauing have not been scientifically proven yet.^20^

## 5 CONCLUSIONS

This fatal case demonstrates that the inappropriate use of anabolising agents has become increasingly widespread in the bodybuilding community. Considering that an acute ingestion/injection rarely leads to poisoning or death, in most of cases, it is long‐term abuse that causes death through cardiac damage or cancer. In this case, the concentration of clenbuterol in the blood and of drostanolone in the urine demonstrates a recent administration, with the achievement of toxic levels of clenbuterol in blood that, in association with a repeated use of multiple other AAS documented by hair results, led to death through cardiac, hepatic and metabolic toxicity. Indeed, the macroscopic and the microscopic analysis revealed multi‐organ congestion involving veins and abdominal organs such the liver and spleen. Furthermore, from the same investigation emerged a massive pulmonary edema with haemorrhagic extravasation and a cardiomegaly with nonobstructive concentric intimal hyperplasia. The results from the hair analysis, considering the ways of incorporating the active principle from the blood to the keratin matrix, the hair growth rate, the window of detectability and the concentrations reported in the literature, support the diagnosis of long‐term abuse of anabolising agents. The lack of a traumatic agent or of other diseases able to explain the death is useful to demonstrate that the only reasonable cause is the one discussed above. This case is remarkable as only a few other case reports in the literature deal with the combined analysis of clenbuterol and anabolic steroids, although these drugs are abused by millions of subjects. On this occasion, the toxicological results overlay the circumstantial data in identifying the cause of death, providing a comprehensive picture of what happened in the previous months of the subject's life.

## Supporting information


**Supplementary Table 1.** Selected analytes, GC retention times and selected ions for GC‐MS analysis of AAS and metabolites in urine.

## Data Availability

Data available on request due to privacy/ethical restrictions.
