# Supplementary material for: Post‐mortem investigation into a death involving doping agents: The case of a body builder
Source: Drug Test Anal. 2022 Aug 14;14(10):1795–9. doi: 10.1002/dta.3350 (PMC9804486; doi:10.1002/dta.3350)
Supplement: Supplementary file 1 — Supplementary Table 1. Selected analytes, GC retention times and selected ions for GC‐MS analysis of AAS and metabolites in urine. [file DTA-14-1795-s001.docx]

| **Analyte** | **Rt**  **(min)** | **rRt** | **Selected ions for multiple ion monitoring, *m/z*** | | | |
| --- | --- | --- | --- | --- | --- | --- |
|  |  |  | Q1 | Q2 | Q3 | Q4 |
| **Salbutamol** | 9.43 | 0.701 | 440 | 369 | 86 | - |
| **Clenbuterol** | 9.83 | 0.731 | 335 | 300 | 86 | - |
| **19-Norandrosterone**  **(nandrolone metabolite 1)** | 11.80 | 0.877 | 405 | 315 | 225 | - |
| **Boldenone metabolite 1** | 11.88 | 0.884 | 432 | 417 | 194 | - |
| **19- Norethiocolanone**  **(nandrolone metabolite 2)** | 12.08 | 0.898 | 405 | 315 | 225 | - |
| **2**$\boldsymbol{\alpha}$**-methyl-5**$\boldsymbol{\alpha}$**-androstan-3**$\boldsymbol{\alpha}$**-ol-17-one (drostanolone metabolite)** | 12.43 | 0.925 | 448 | 433 | 343 | - |
| $\boldsymbol{1}\boldsymbol{\alpha}$**-methyl-5**$\boldsymbol{\alpha}$**-androstan-3**$\boldsymbol{\alpha}$**-ol-17-one (mesterolone metabolite)** | 12.72 | 0.946 | 448 | 433 | 343 | - |
| $\boldsymbol{17}\boldsymbol{\alpha}$**-methyl-5**$\boldsymbol{\alpha}$**-androstan-3**$\boldsymbol{\alpha}$**-17**$\boldsymbol{\beta}$**-diol (methyltestosterone metabolite 1)** | 12.74 | 0.945 | 450 | 435 | 345 | 255 |
| $\boldsymbol{17}\boldsymbol{\alpha}$**-methyl-5**$\boldsymbol{\alpha}$**-androstan-3**$\boldsymbol{\alpha}$**-17**$\boldsymbol{\beta}$**-diol (methyltestosterone metabolite 2)** | 12.74 | 0.945 | 450 | 435 | 345 | 255 |
| **Epitestosterone** | 12.76 | 0.949 | 432 | 417 | - | - |
| **Boldenone** | 12.92 | 0.961 | 430 | 206 | 325 | - |
| $\boldsymbol{7\beta,}\boldsymbol{17}\boldsymbol{\alpha}$**-dimethyl-5**$\boldsymbol{\beta}$**-androstan-3**$\boldsymbol{\alpha}$**-17**$\boldsymbol{\beta}$**-diol (calusterone metabolite)** | 12.98 | 0.966 | 374 | 284 | 269 | - |
| **Testosterone** | 13.01 | 0.968 | 432 | 417 | - | - |
| **Oxandrolone** | 13.67 | 1.017 | 378 | 363 | 308 | - |
| **6**$\boldsymbol{\beta}$**-hydroxymethandienone (metandienone metabolite )** | 14.02 | 1.043 | 532 | 517 | 427 | 294 |
| **Fluoxymesterone** | 14.23 | 1.058 | 552 | 462 | 447 | - |
| **Oxymesterone** | 14.29 | 1.063 | 519 | 444 | 389 | - |
| **6-**$\boldsymbol{\beta}$**Hydroxyfluoxymesterone (fluoxymesterone metabolite)** | 14.84 | 1.104 | 642 | 640 | 625 | - |
| **16-**$\boldsymbol{\beta}$ **hydroxyfurazabole (furazabole metabolite)** | 15.21 | 1.132 | 490 | 231 | 218 | - |
| **16-**$\boldsymbol{\beta}$ **hydroxystanozolol (metabolite stanozolol 1)** | 15.34 | 1.141 | 560 | 545 | 455 | 254 |
| **3’-hydroxystanozolol (metabolite stanozolol 2)** | 15.34 | 1.141 | 560 | 545 | 455 | 254 |
| **Methyltestosterone (IS1)** | 13.44 | 1.000 | 436 | 356 | 301 | - |
| **Norethiocolanone-d4 (IS2)** | 12.06 | 0.897 | 409 | 319 | 229 | - |

**Rt = Retention time, rRt= relative retention time (Rt/Rt IS1), IS = internal standard.**

**Supplementary Table 1. Selected analytes, GC retention times and selected ions for GC-MS analysis of AAS and metabolites in urine.**
